# Pediatric central nervous system tumor with *CIC::LEUTX* fusion: a diagnostic challenge

**DOI:** 10.1186/s40478-024-01824-w

**Published:** 2024-06-27

**Authors:** Yanghao Hou, Yanru Du, Juan Wang, Xinke Zhang, Xueyan Zhao, Xinyi Xian, Li Yuan, Haigang Li, Yu Wang, Shaoyan Xi, Guan Huang, Wenbiao Zhu, Juan Wang, Jin Zhu, Qiubo Yu, Youde Cao, JingXian Wu, Jing Zeng, Gehong Dong, Wanming Hu

**Affiliations:** 1 Department of Pathology, Center for Molecular Medicine Testing, College of Basic Medicine, Chongqing Medical University, Chongqing, P. R. China; 2 Department of Pathology, State Key Laboratory of Oncology in South China, Guangdong Provincial Clinical Research Center for Cancer, Sun Yat-sen University Cancer Center, Guangzhou, P. R. China; 3 Department of Pathology, Beijing Tiantan Hospital, Capital Medical University, Beijing, P. R. China; 4 Department of Pathology, Nanjing Brain Hospital, Nanjing, P. R. China; 5 Department of Pathology, The First Affiliated Hospital of Zhengzhou University, Zhengzhou, P. R. China; 6 Department of Pathology, Guangzhou Women and Children Medical Center, Guangzhou, P. R. China; 7 Department of Pathology, Sun Yat-sen Memorial Hospital, Guangzhou, P. R. China; 8 Department of Pathology, Zhujiang Hospital of Southern Medical University, Zhujiang, P. R. China; 9 Department of Pathology, Longgang District Central Hospital of Shenzhen, Shenzhen, P. R. China; 10 Department of Pathology, Meizhou People’s Hospital, Meizhou, P. R. China; 11 Department of Pediatric tumor, State Key Laboratory of Oncology in South China, Guangdong Provincial Clinical Research Center for Cancer, Sun Yat-sen University Cancer Center, Guangzhou, P. R. China; 12 Department of Pathology, Children’s hospital of Chongqing medical university, Chongqing, P. R. China

## Introduction

The fifth edition of the World Health Organization Classification of Tumors of the Central Nervous System (WHO CNS5) recognized *CIC*-rearranged sarcoma as a distinct mesenchymal, non-meningothelial tumor, designated as CNS WHO grade 4 [1, 2]. This entity is characterized by its *CIC* gene fusion with various partners, most notably *NUMT1* or *DUX4* [3, 4], serving as crucial molecular hallmarks and essential criterion for diagnosis [1, 4]. However, the genetic landscape, etiology and clinical implications of *CIC*-fused CNS tumors still remain elusive, posing significant challenges in routine diagnostic practices.

Recent studies have identified a set of pediatric high-grade neuroepithelial tumors with *CIC* fusions, which display a unique DNA methylation profile differing from *CIC*-rearranged sarcomas, suggesting an intermediate malignancy grade [5]. These findings elucidate that CNS tumors harboring *CIC* gene fusions may encompass various tumor types and divergent clinical outcomes.

DNA methylation profiling has emerged as a robust and dominant approach for the comprehensive classification of CNS tumors and the identification of new tumor entities [6–10]. WHO CNS5 also recommends this technology as a desirable tool for diagnosis, particularly in pediatric and embryonal tumors [1, 11–13]. Given the potential of *CIC* fusions to occur beyond CNS sarcomas, integrating methylation profiling is crucial for distinguishing other tumor entities that share *CIC* alterations.

In this multicenter study, we describe a cohort of tumors with *CIC* fusions, including 14 CNS cases (containing 6 *CIC::LEUTX* fusion tumors) and 5 peripheral sarcoma cases, with a particular focus on those with rare fusion partners and their diverse clinical outcomes. These tumors underwent comprehensive histological re-evaluation, RNA sequencing, DNA sequencing and genome-wide methylation profiling. Our aim is to deepen and broaden the clinical, histological, and molecular understanding of *CIC*-fused CNS tumors, as well as to assess whether CNS tumors with some specific *CIC* fusions should be considered as a distinct entity.

## Materials and methods

### Sample collection

Tumor specimens with *CIC* fusions were acquired from: the department of pathology of Sun Yat-sen University Cancer Center, Children’s hospital of Chongqing medical university, Guangzhou Women and Children Medical Center, Beijing Tiantan Hospital, Zhujiang Hospital of Southern Medical University, Nanjing Brain Hospital, Sun Yat-sen Memorial Hospital, Longgang District Central Hospital of Shenzhen, Meizhou People’s Hospital and the First Affiliated Hospital of Zhengzhou University. The whole series comprised 14 CNS tumors with *CIC* fusions including 6 cases each of *CIC::LEUTX* and *CIC::NUTM1*, one case of *CIC::FOXO4*, and one case of concurrent *CIC::DUX4* & *CIC::FRG2B*, and 5 cases of peripheral *CIC* sarcomas, consisting of 3 cases of *CIC::DUX4* (one of which also featured a *CIC::DBET* intergenic region fusion), one case of *CIC::FBXO4*, and one case of a *CIC::LINC00854-LINC00910* intergenic region fusion. Reference group for methylation profiling was selected from Gene Expression Omnibus (GEO) database, specifically datasets GSE90496 and GSE223546. The collection of tumor samples and clinical data were processed in an accordance with standards approved by the ethical committees of department of pathology and center for molecular medicine testing, Chongqing medical university.

### Histological analysis

Histological assessments were evaluated by three neuropathologists (Wanming Hu, Jing Zeng and Yanghao Hou) following the diagnostic guidelines of CNS WHO 5. Immunohistochemistry (IHC) staining was performed on 3 μm-thick formalin-fixed, paraffin-embedded (FFPE) sections using an automated BenchMark Ultra (Ventana Medical systems, Roche, SW). Antibodies were diluted against: GFAP (ZSGB-BIO, 1:200), Olig-2 (ZSGB-BIO, 1:200), synaptophysin (ZSGB-BIO, 1:200), WT-1 (ZSGB-BIO, 1:200), CD99 (ZSGB-BIO, 1:200), Ki67 (DAKO, RTU). Special reticulin staining was used VENTANA Silver ISH DNP Detection Kit.

### DNA and RNA extraction

Areas rich in tumor cells (> 70% tumor cell content) were identified on hematoxylin and eosin (H&E) stained slides. Necrotic and lymphocyte-rich areas was avoided to ensure the quality of methylation array and next generation sequencing. DNA and RNA were obtained separately from 10 individual 10 μm-thick FFPE sections, with areas precisely matched the selected regions identified by H&E staining to ensure optimal tumor cell content and quality for molecular analysis. Extractions were performed using QIAamp DNA FFPE Tissue kit (QIAGEN, Germany) and RNeasy FFPE kits (QIAGEN, Germany), according to the manufacturer’s protocols.

### DNA methylation and copy number profiling

The raw DNA methylation data (.idat) were obtained from the Infinium MethylationEPIC (850 K) or Infinium MethylationEPIC2.0 (935 K) BeadChip array (Illumina, San Diego, USA), DNA extracted from FFPE tissue were all repaired by Infinium FFPE QC and DNA Restoration Kits (WG-321-1002, Illumina, San Diego, USA) following the manufacturer’s instructions as previously described [14]. BeadChip were scanned by the iScan (Illumina, San Diego, USA). Raw idat files were proceeded in R by package “minfi” and “sesame”, “limma” package was used to remove batch effect. The same probe that preserved in both 450k (reference set), 850k (reference set and local sample set) and 935k (local sample set) were selected for beta-value calculation. In total, five samples failed to perform methylation array due to insufficient DNA quantity. Copy number profiling were derived from the methylation raw data in R version 4.3 (https://www.R-project.org) by using the package “conumee” (http://bioconductor.org/packages/conumee/).

### Next generation sequencing

A panel-based NGS assay was used to detect gene alterations in formalin-fixed, paraffin-embedded (FFPE) tumor samples. First, DNA was extracted from undyed FFPE sections with the proportion of tumor cells more than 20% and whole-blood samples and then purification and library preparation were performed. Second, a probe with 2.29 Mbp in size was used for hybrid capture and enrichment in gene-specific regions where various aberrations of 360 cancer-related genes including single nucleotide variants, copy number variations, small insertions, deletions and gene arrangements were covered. R package “maftools” was used for generating tumor mutation plots.

### RNA sequencing and fusion calling

The quality of each RNA sample was tested using Qubit 4.0 and Agilent 4200 TapeStation system prior to library preparation and sequencing. cDNA synthesis, and library preparation were performed using the KAPA RNA HyperPrep Kit (Kapa Biosystems, KK8540). The total volume of the final library was at least 40 ng. No obvious joint contamination was detected in the final library using the Agilent 4200 TapeStation system, and the main peak was between 300 and 500 bp. After quantification, NGS was performed on an Illumina Novaseq 6000 instrument (Illumina).

The software fastp (v.2.20.0) was used for adapter trimming. The software STAR (v2.7.6a) was used to align reads to the reference genome (UCSC’s hg19 GRCh37). Fusion expression was calculated based on fusion fragment per million (FFPM) using the raw data from the RNA fusion panel (166 genes). We use STAR-Fusion software (v1.9.1) to perform fusion detection. The coverage of gene exons for probe was calculated based on the fragments per kilobase of exon model per million mapped fragments (FPKM).

### Statistical analysis

For T-Distributed Stochastic Neighbor Embedding (t-SNE) and unsupervised hierarchical clustering analysis, 15,000 most variable CpG sites with the highest median absolute deviation were been selected. 2500 iterations and a perplexity value of 5 was configured for t-SNE plotting. Graphic visualizations were conducted by R packages “ggplot2”. Displaying reads of fusion genes using the IGV (The Integrative Genomics Viewer, https://igv.org/doc/desktop/) tool. Kaplan–Meier survival curves was performed using R packages “survival” and “surminer”.

## Results

### Methylation signature of *CIC*-fused tumors

**Fig. 1:**
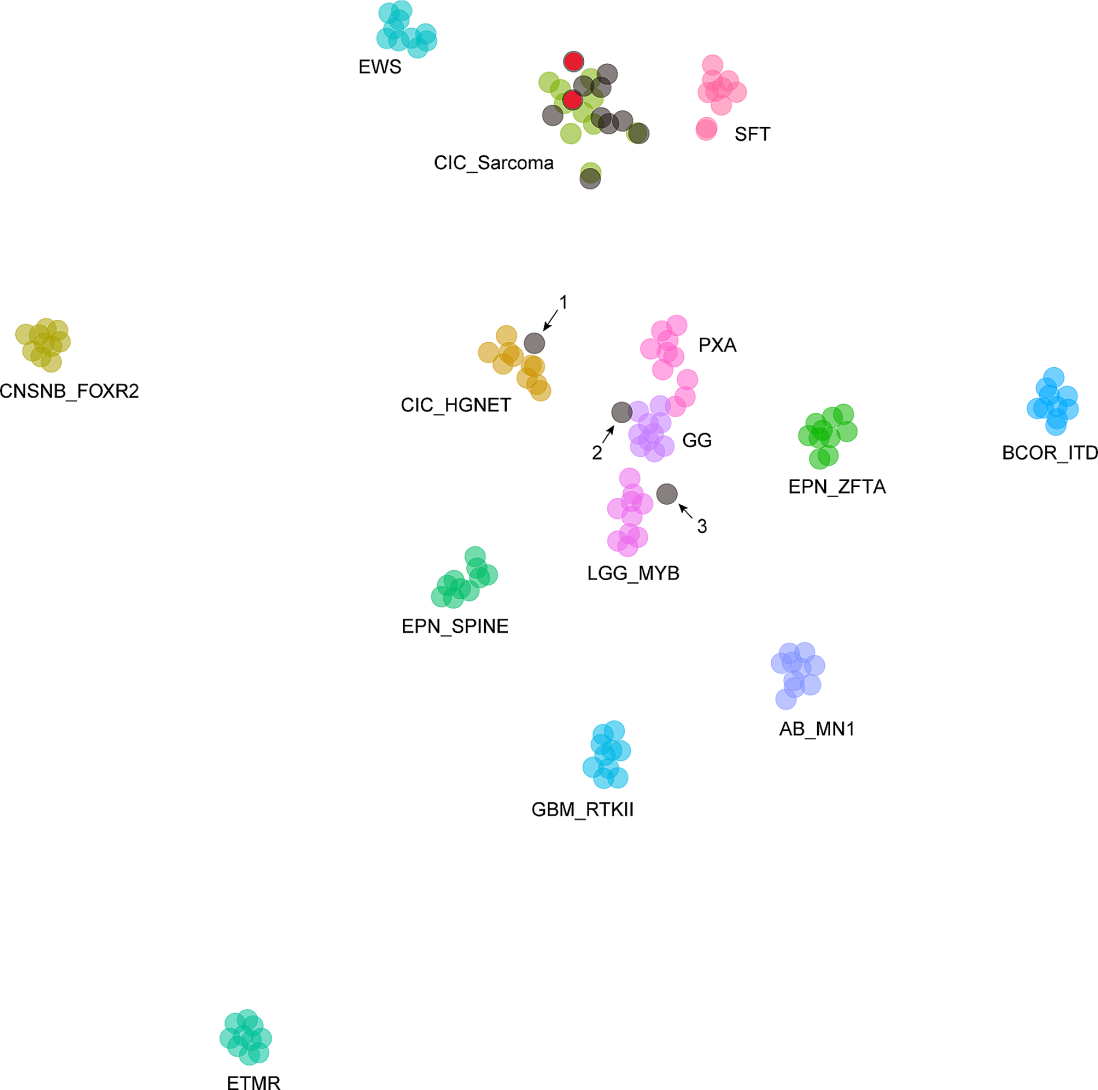
Methylation based t-distributed stochastic neighbor embedding (t-SNE) analysis of 12 *CIC*-fused CNS tumours (colored in gray) and 2 peripheral tumors harboring *CIC::DUX4* fusion (colored in red). All cases were clustered to conventional *CIC*-altered sarcoma except for case 1, case 2 and case 3 with *CIC::LEUTX* fusion. *AB_MN1* MC Astroblastoma *MN1*-altered, *BCOR_ITD* MC CNS tumour with *BCOR* internal tandem duplication, *CIC_Sarcoma* MC *CIC*-altered sarcoma, *CNSNB_FOXR2* MC CNS neuroblastoma *FOXR2*-activated, *CIC_HGNET* MC high-grade neuroepithelial tumor *CIC* fusion-positive, *EWS* MC Ewing sarcoma, *EPN_SPINE* MC spinal ependymoma, *EPN_ZFTA* MC ependymoma ZFTA fusion-positive, *ETMR* MC Embryonal tumour with multilayered rosettes, *GG* MC Ganglioglioma, *GBM_RTKII* MC glioblastoma RTKII subtype, *LGG_MYB* MC diffuse astrocytoma *MYB* altered, *PXA* MC pleomorphic xanthoastrocytoma, *SFT* MC solitary fibrous tumor

We performed t-SNE analysis by using our cohort (*n* = 14) together with 1889 published reference cases [7], encompassing nearly all CNS tumor types and subtypes, including the recently published methylation class (MC): high-grade neuroepithelial tumor (HGNET) *CIC* fusion-positive [5]. We further refined t-SNE analysis for better visualization (Fig. 1) by narrowing representative MC, the clustering results of each case were consistent with the broader t-SNE analysis.

Of the 12 CNS tumors with various *CIC* fusion partners, nine cases clustered with the reference MC: *CIC*-rearranged sarcoma, alongside 2 peripheral sarcomas with *CIC::DUX4* gene fusion, serving as internal controls. Notably, three of the six CNS tumors with *CIC::LEUTX* fusions clustered elsewhere: Case 1 clustered to the reference set “HGNET *CIC* fusion-positive”. Case 2 clustered closely to the MC ganglioglioma, and Case 3 exhibited an independent methylation signature near the reference methylation group for low-grade glioma, *MYB*-altered.

### Invariable *LEUTX* locus aberrations on chromosome 19

**Fig. 2:**
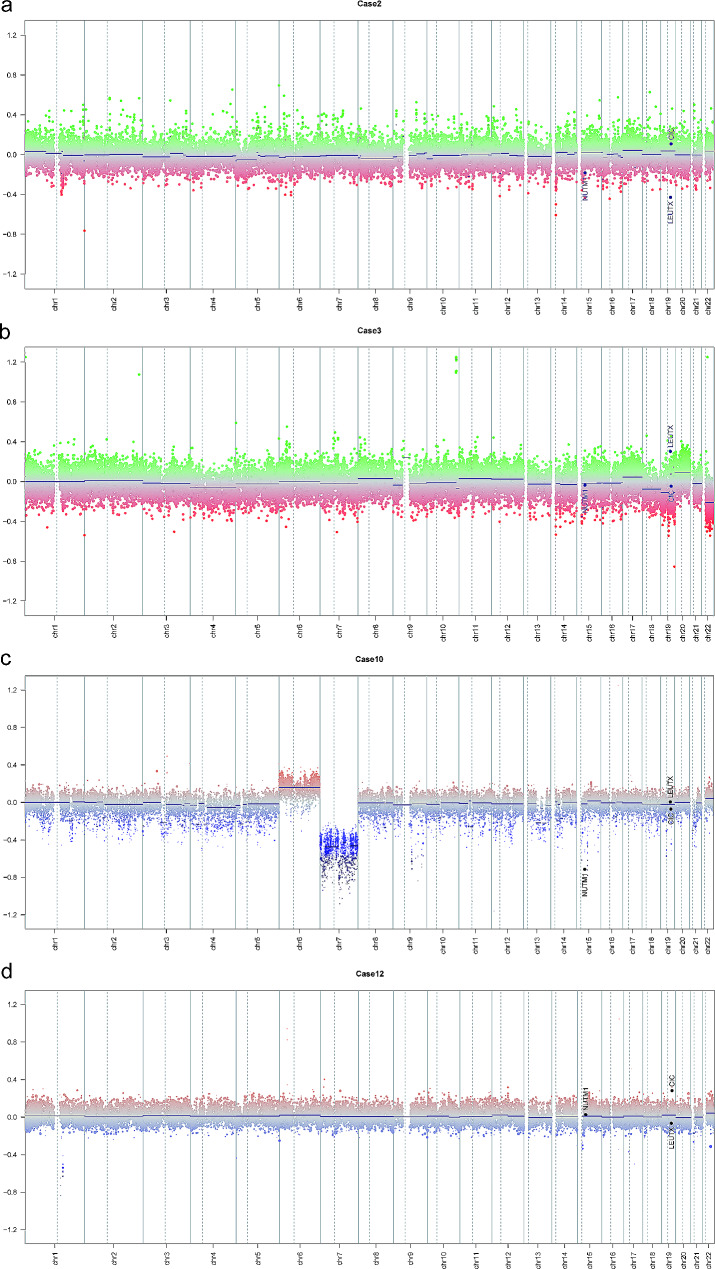
Representative copy-number profiles derived from methylation data. Loss **(a)** and gain **(b)** were observable at the *LEUTX* locus (19q13.2) on chromosome 19q. Loss of *NUTM1* (15q14) in case 10 with *NUTM1::CIC* fusion **(c)**. Gain of CIC locus (19q13.2) in Case 12 harboring *CIC::FOXO4* fusion **(d)**

In all six primary CNS tumors featuring *CIC::LEUTX* gene fusions, methylation array-based copy number profiling consistently revealed *LEUTX* locus aberrations at chromosome 19q13.2, demonstrated by visual inspection of *LEUTX* locus loss or gain (6/6, 100%) in CNV results (Fig. 2a, b). Among the tumors with *CIC::NUTM1* fusion, alterations were observed at the *NUTM1* locus (15q14) in two cases and at the *CIC* locus (19q13.2) in one case (Fig. 2c). Case 12 (with *CIC::FOXO4* fusion) and Case 18 (*CIC* intergenic rearrangement) exhibited aberrations at the *CIC* locus (Fig. 2d). Furthermore, gains on chromosome 8 (4/14, 28%) were frequently observed. Detailed descriptions of these chromosome changes are provided in Table 1.

**Table 1:** Molecular characteristics of 19 *CIC*-fused tumors

No	Fusion	Methylation class	locus change	Chromosome
Case 1	*CIC::LEUTX*	*CIC*_HGNET	*LEUTX* loss	Gain: 1q,2,3,6,8,12,14,17,19,20,21; Loss: 10
Case 2	*CIC::LEUTX*	near GG	*LEUTX* loss	Flat
Case 3	*CIC::LEUTX*	Undefined	*LEUTX* gain	Loss: 22q
Case 4	*CIC::LEUTX*	*CIC*_Sarcoma	*LEUTX* loss	Gain: 8,12,17,19; Loss: 13q
Case 5	*CIC::LEUTX*	*CIC*_Sarcoma	*LEUTX* loss	Flat
Case 6	*CIC::LEUTX*	*CIC*_Sarcoma	*LEUTX* gain	Gain: 1q, partial 2q, 19.
Case 7	*CIC::NUTM1*	*CIC*_Sarcoma	*CIC* gain	Gain: 5, 8, 16, 17, 21q
Case 8	*CIC::NUTM1*	*CIC*_Sarcoma	*NUTM1* gain	Loss: 19p
Case 9	*CIC: NUTM1*	N/A	N/A	N/A
Case 10	*CIC::NUTM1*	*CIC*_Sarcoma	*NUTM1* loss	Gain: 6; Loss: 7
Case 11	*CIC::DUX4*	*CIC*_Sarcoma	N/A*	Gain: 19, 22q
Case 12	*CIC::FOXO4*	*CIC*_Sarcoma	*CIC* gain	Flat
Case 13	*CIC::NUTM1*	*CIC*_Sarcoma	None	Flat
Case 14	*CIC::NUTM1*	N/A	N/A	N/A
Case 15	*CIC-DUX4*	*CIC*_Sarcoma	None	Gain: 8,19; Loss: 22q
Case 16	*CIC-DUX4*	N/A	N/A	N/A
Case 17	*CIC-FBXO4*	N/A	N/A	N/A
Case 18	*CIC-DUX4*	*CIC*_Sarcoma	*CIC* gain	Gain: partial 1q,6,18,22; Loss: partial 1p, partial 1q,7,13q
Case 19	*CIC-intergenic rearrangement*	N/A	N/A	N/A

*CNV influenced by low DNA quality

### Clinical findings

The clinical data for the cohort are detailed in Table 2. Of the 14 patients with *CIC*-fused CNS tumors, 8 were male and 6 were female, with a median age at diagnosis of 5 years (range:1–17). The tumors manifested across various brain regions and parts of the spinal cord, most commonly in cerebral hemispheres.

**Table 2:** Clinical findings of 19 tumors harboring *CIC* fusions

No	Age	Sex	Location	Histology	Fusion	Therapy	Follow-up (mo)	Statues
Case 1	4y	M	Left temporal lobe	HGNET; pHGG, IDH/H3-wt	*CIC-LEUTX*	GTR + TMZ+ RT	48 (2nd relapse)	Alive
Case 2	2y	M	Left temporal lobe-basal ganglia and left parietal lobe	HGNET; Embryonal Tumor, NOS	*CIC-LEUTX*	GTR + PR + RT + everolimus	56 (Surviving with stable post- surgical enhancement)	Alive
Case 3	9y	M	Left occipital lobe	Low-grade NET; PXA	*CIC-LEUTX*	GTR	34	Alive
Case 4	3y	F	Left temporo-occipital	CNS tumor, NOS	*CIC-LEUTX*	N/A	N/A	N/A
Case 5	5y	F	Brainstem	CNS tumor, NOS	*CIC-LEUTX*	STR	6	Alive
Case 6	16y	M	Spinal cord	CNS tumor, NOS	*CIC-LEUTX*	N/A	N/A	N/A
Case 7	1y	M	Left ventricle and left parietal lobe	CNS tumor, NOS	*CIC-NUTM1*	GTR + TMZ	1	DOD
Case 8	3y	M	Right parieto-occipital lobe	Embryonal tumor, NOS	*CIC-NUTM1*	GTR	N/A	N/A
Case 9	5y	M	Left occipital lobe	Undifferentiated Small Round Cell tumor	*CIC-NUTM1*	GTR + RT(60 Gy/30F/2Gy)	7	N/A
Case 10	6y	F	Right temporoparietal (intracranial and extracranial)	Sarcoma	*CIC-NUTM1*	GTR + CAV/IE + L-MTX + VI	21 (metastasis to right iliac wing)	Alive
Case 11	9y	M	Frontal lobe	PNET, NOS	*CIC-DUX4*	GTR + EP/CTX + CBP + VCR	17	DOD
Case 12	10y	F	Fourth ventricle	Small Blue Round Cell Tumor	*CIC-FOXO4*	GTR	1	DOD
Case 13	11y	F	C7 to T2 spinal cord	Small Blue Round Cell Tumor	*CIC-NUTM1*	STR	NA	NA
Case 14	17y	F	Lumbar vertebra 4	Sarcoma	*CIC-NUTM1*	Doxorubicin + Ifosfamide	6	DOD
Case 15	26y	M	(peripheral) Abdominal wall	Sarcoma	*CIC-DUX4*	NA	NA	NA
Case 16	32y	M	(peripheral) Scalp and pulmonary metastasis	Sarcoma	*CIC-DUX4*	GTR + CAV/IE	26 (lung metastases)	Alive
Case 17	36y	F	(peripheral) Left cervical-shoulder region	Sarcoma; Ewing-like sarcoma	*CIC-FBXO4*	Enrolled to clinical test	15	Alive
Case 18	40y	M	(peripheral) Right abdominal wall (within the rectus abdominis)	Angiosarcoma	*CIC-DUX4*	Doxorubicin + Ifosfamide	25 (2nd relapse)	NA
Case 19	8y	M	(peripheral) Mass above the left scapula and right hilum right thoracic cavity	Sarcoma; Ewing-like sarcoma	*CIC*-intergenic rearrangement	CTX + THP+ DDP	7	Alive

GRT, gross total resection; STR, subtotal resection ; PR(CTX + CBP + VCR/DDP + VP-16); RT, radiotherapy; TMZ, temozolomide; CAV, Cyclophosphamide, Doxorubicin, and Vincristine; VP-16,Etoposide; IE, Ifosfamide and Etoposide; L-MTX, Liposomal Mitoxantrone; VI, vincristine and Ifosfamide; EP, etoposide; CTX, cyclophosphamide; CBP, carboplatin; VCR, vincristine ; THP, trastuzumab, pertuzumab and taxane; DDP, Cisplatin; DOD, Died of disease; N/A, not available

### Histological and immunohistochemical characteristics of *CIC* and *CIC::LEUTX* fused tumors

**Fig. 3:**
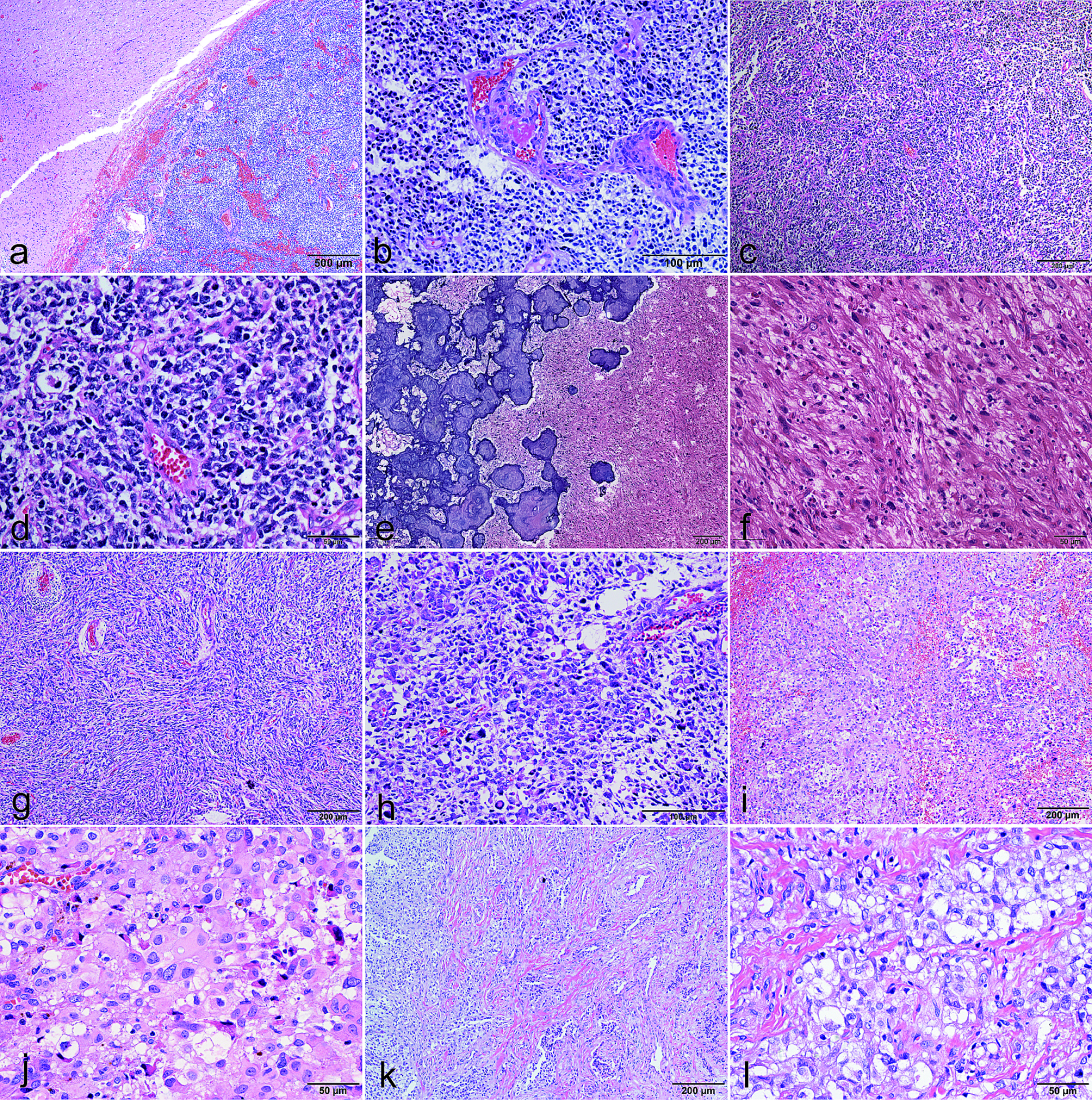
Variable morphological features of *CIC::LEUTX* fused CNS tumors. **Case 1 (a, b)** showed well-defined boundaries from the surrounding brain tissue, and a high-grade neuroepithelial tumor appearance with vascular endothelial hyperplasia; **Case 2 (c, d)** was initially diagnosed as an embryonal tumor due to small round blue cell embryonal tumor morphology; **Case 3 (e, f)** exhibit low-grade morphological features with calcifications and pleomorphic GFAP & Olig-2 positive tumor cells; **Case 4 (g, h)** exhibited spindled to gemistocytic/epithelioid cytology. **Case 5 (i, j)** is characterized by epithelioid tumor cells with prominent nucleolus, mimicking metastatic carcinoma; **Case 6 (k, l)** shows a nodular and diffuse sheeting growth pattern, clear-cell cytology is focally presented

Histological examination of the *CIC*-fused CNS tumors revealed a diverse morphological spectrum (Fig. 3). Typical features included highly undifferentiated, small to medium-sized blue round cells with brisk mitotic activity, microvascular proliferation, and necrosis. A range of cellular morphologies, including gemistocytic, epithelial, giant, triangular and spindle cells, was noted across different cases, with perivascular pseudorosette occasionally observed.

**Fig. 4:**
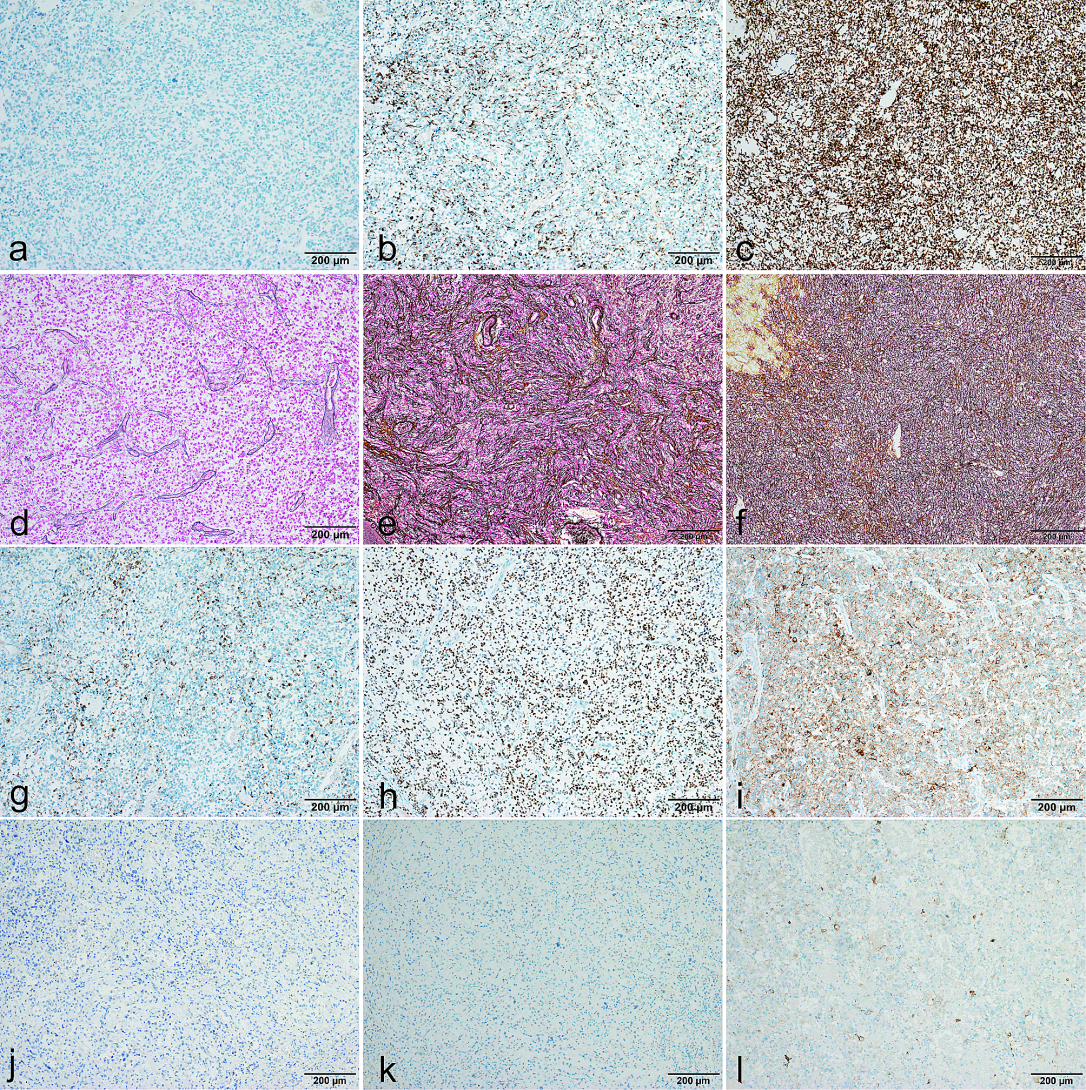
Representative immunohistochemistry and reticulin staining. Total negativity of WT-1 staining in Case 1 **(a)** in comparison to focal positivity in Case 4 **(b)** and diffuse WT-1 expression in non-*LEUTX CIC*-rearranged tumors **(c, Case 8)**; Absence of reticular fibers in Case 1 **(d)** in comparison to rich reticulin staining in Case 4–19 **(e, Case 4; f, Case 8);** GFAP, Olig-2 and Synaptophysin expression of **Case 1 (g, h, i) and Case 5 (j, k, l)**

In tumors harboring the *CIC::LEUTX* fusion, a glial fibrillary matrix could be found, with robust expression of glial markers (GFAP, Olig-2) and neuronal markers (synaptophysin). Notably, two distinct immunophenotypic patterns emerged. One was a neuroepithelial tumor pattern (Case 1–3) characterized by a lack of WT-1 expression (Fig. 4a) and absence of reticular fibers, except in perivascular spaces (Fig. 4d). The other pattern, resembling sarcomas (Case 4–6), showed positive WT-1 staining (Fig. 4b) and reticular fibers encircling individual cells (Fig. 4e), similar to peripheral *CIC* sarcomas (Fig. 4c, f). Detailed IHC results are provided in Table 3.

**Table 3:** Immunohistochemistry results

No.	GFAP	Olig2	Syn	WT1	Reticulin	CD99	Ki67
Case 1	**±**	**+**	**±**	**-**	**-**	**±**	**50%**
Case 2	**-**	**-**	**+**	**-**	**-**	**±**	**50%**
Case 3	**+**	**±**	**-**	**-**	**-**	**N/A**	**1%**
Case 4	**-**	**-**	**-**	**±**	**+**	**N/A**	**30%**
Case 5	**-**	**-**	**±**	**±**	**+**	**±**	**30%**
Case 6	**±**	**N/A**	**±**	**±**	**+**	**+**	**20%**
Case 7	**±**	**+**	**-**	**±**	**+**	**N/A**	**30%**
Case 8	**-**	**-**	**-**	**+**	**+**	**+**	**50%**
Case 9	**-**	**-**	**±**	**+**	**+**	**±**	**70%**
Case 10	**-**	**-**	**-**	**±**	**+**	**N/A**	**60%**
Case 11	**-**	**-**	**-**	**N/A**	**+**	**+**	**50%**
Case 12	**-**	**-**	**-**	**+**	**+**	**+**	**70%**
Case 13	**-**	**-**	**-**	**N/A**	**+**	**±**	**90%**
Case 14	**-**	**-**	**-**	**±**	**+**	**-**	**15%**
Case 15	**-**	**-**	**-**	**+**	**+**	**+**	**70%**
Case 16	**-**	**-**	**-**	**+**	**+**	**+**	**30%**
Case 17	**-**	**-**	**-**	**±**	**+**	**+**	**80%**
Case 18	**-**	**-**	**-**	**±**	**+**	**+**	**80%**
Case 19	**-**	**-**	**-**	**+**	**+**	**+**	**60%**

Positive is documented as “+”, focal as “±”, and absent as “−”; N/A: not available

The detailed description of 3 *CIC::LEUTX* fused CNS tumors (not aligned to MC *CIC*-rearranged sarcoma in methylation profiling) were listed below.

### Case 1 (Fig. 3a, b)

Histologic examination revealed a high-grade neuroepithelial tumor characterized by densely packed, poorly differentiated cells displaying nuclear atypia and marked pleomorphism. The mitotic activity is brisk, accompanied by prominent microvascular proliferation and necrosis. IHC showed focal GFAP expression (Fig. 4g) and strong positivity for Olig-2 (Fig. 4h) and synaptophysin (Fig. 4i). WT-1 was negative (Fig. 4a). Additionally, reticulin staining revealed a pattern typical for neuroepithelial tumors, with reticular fibers confined to vascular areas and absent within the tumor parenchyma (Fig. 4d). These morphological features were consistent with a malignant glioma of WHO grade 4.

### Case 2 (Fig. 3c, d)

The tumor also presented as a high-grade neuroepithelial tumor. It consisted of poorly differentiated or undifferentiated cells interspersed with neuropil-like structures and abundant vascular proliferation. No typical true rosettes were found. Some tumor cells were small, round, and poorly differentiated, whereas others displayed atypia and rough, dark chromatin. Mitotic figures and apoptotic bodies were readily apparent under HPF. IHC showed negative staining for both GFAP and Olig-2. WT-1 was also negative. Reticular staining did not reveal significant desmoplasia but maintained a pattern consistent with neuroepithelial tumors. Remarkably, the tumor cells exhibited strong positivity for synaptophysin. The preliminary diagnosis was CNS embryonal tumor, NOS, supported by morphology and its strong synaptophysin immunoreactivity. This case was previously described and published as a case study [15] but without methylation analysis.

### Case 3 (Fig. 3e, f)

The tumor specimens documented spindle cells and large pleomorphic cells with lipidized cytoplasm, alongside notable calcification, suggesting a low-grade glioma. The tumor cells were positive for both GFAP and olig-2. Staining for Synaptophysin, BRAF, and H3K27M were negative. ATRX and INI1 were retained. The Ki67 proliferation index was only 1%, and WT-1 was also negative. In summary, it displayed a low-grade tumor morphological appearance which could potentially mimic pleomorphic xanthoastrocytoma (PXA) or polymorphous low-grade neuroepithelial tumour of the young (PLNTY), However, unlike PXA or PLNTY, reticular fibers were only observed surrounding blood vessels, as well as the CD34 expression. It also did not exhibit *BRAF V600E* mutations or *CDKN2A/B* deletions. The primary diagnosis was PXA-like LGG, NEC.

Notably, different from peripheral *CIC* sarcomas, the nucleoli of the tumor cells in the above three cases were not obvious.

### Case 4 (Fig. 3g, h), 5 (Fig. 3i, j) and 6 (Fig. 3k, l)

These three cases were *CIC::LEUTX* fused CNS tumors which clustered to MC *CIC*-rearranged sarcoma in methylation profiling, Their morphology was also similar to that of peripheral *CIC* sarcoma. The tumor displayed variable cell morphology ranging from spindled to gemistocytic/epithelioid, including sheets of monotonous cells with high nuclear: cytoplasmic ratios and distinct nucleoli. Some areas showed high cellularity with uniform, round to oval nuclei, while large nucleoli were prominent in these tumor cells. Areas resembling fibro/sarcomatoid spindle cells may also be seen, which looks more like a sarcoma. Case 5 even looked like angiosarcoma, for there was a significant presence of small blood vessel proliferation, hemorrhage, and red blood cell exudation. The tumor cells were negative for GFAP (Fig. 4j), Olig-2 (Fig. 4k), and only individual tumor cells expressed synaptophysin (Fig. 4l). WT-1 showed focal positive expression (Fig. 4b), but not diffusely strong as other non-*LEUTX*::*CIC-*rearranged tumors (Fig. 4c). Reticular fibers were abundant (Fig. 4e) and distinct from case 1–3 (Fig. 4d), with more fibers encircling individual cells, similar to the pattern observed in peripheral *CIC* sarcoma (Fig. 4f).

## Discussion

In our study of 19 tumors featuring *CIC* fusions, spanning both CNS and peripheral tissues, we observed that only CNS tumors harboring the *CIC::LEUTX* fusion exhibited neuroepithelial differentiation with better outcomes compared to those of *CIC*-rearranged sarcomas. The distinct diagnostic features for these tumors included positive GFAP and Olig-2 expression, negativity of WT-1 and reticulin, and a methylation profile incompatible with conventional MC *CIC*-rearranged sarcomas.

The *LEUTX* gene, also known as Leucine Twenty Homeobox, is integral to embryonic development and early cellular differentiation [16]. Recent research has linked gene fusions involving *LEUTX* to oncogenesis, particularly in primary CNS sarcomas and high-grade neuroepithelial tumors that harbor *CIC::LEUTX* fusions [5, 17], as well as in embryonal tumors with *BRD::LEUTX* fusions [18]. These rare entities, especially affecting young children, typically exhibit poor clinical outcomes. Notably, within our cohort, we discovered two cases of neuroepithelial tumors with *CIC::LEUTX* fusion that demonstrated prolonged progression-free survival (PFS) and presented with unique, unassigned methylation signatures. This includes one case with low-grade morphological features which have not been previously reported in *CIC*-fused CNS tumors.

### Diagnostic implications for tumors harboring *CIC* fusion

The variable morphological features of pediatric CNS tumors with *CIC* fusions frequently pose diagnostic challenges. Nonetheless, molecular evidence of a *CIC* fusion (the first essential diagnostic criteria of *CIC*-rearranged sarcoma listed by WHO CNS5) or a methylation profile matched to MC *CIC*-sarcoma generally confirms the diagnosis [1].

Our study reveals that pediatric CNS tumors harboring the *CIC::LEUTX* fusion represent a heterogeneous set of tumors. These occur across a spectrum including conventional *CIC*-altered sarcoma, high-grade neuroepithelial tumor, and even rare lower-grade glial tumors, indicating that not all the intracranial *CIC*-fused tumors are *CIC*-altered sarcomas, especially those with *CIC::LEUTX* genetic fusion. In contrast to peripheral *CIC*-rearranged sarcomas, which typically express WT-1, lack GFAP and Olig-2, and have abundant reticular fibers [19], intracranial *CIC::LEUTX* fused tumors may be considered as neuroepithelial tumors if they exhibit following features, especially the last two items: (1) GFAP & Olig-2 expression (at least focally positive); (2) Synaptophysin expression; (3) Absence of WT-1 expression; (4) Lack of reticular fibers. Given our limited cohort, these observations should be seen as preliminary and indicative rather than definitive criteria.

For unresolved cases, DNA methylation profiling has proven to be the most effective molecular approach for the precise classification of *CIC*-fused CNS tumors [20]. The diagnosis can be assigned if the methylation profile aligns to its representative MC: *CIC*-rearranged sarcoma [7] or the novel entity “HGNET *CIC* fusion positive” [5]. If the methylation result is paradoxical to their morphological or molecular features, as seen in case 2 and case 3 in our cohort, it remains debatable to classify these tumors as ganglioglioma and LGG *MYB*. However, these cases have demonstrated PFS of 56 and 34 months, respectively, despite their *CIC* alterations and mild to aggressive morphological appearances.

Additionally, methylation derived copy-number profiling can provide crucial evidence of *CIC*-related fusions. In our series, 10 out of 12 CNS tumors showed gene locus aberrations related to their fusion partners, particularly those with *LEUTX*, where all six cases demonstrated *LEUTX* locus aberrations.

### Clinical values of subclassification *CIC*-fused tumors

The distinction between *CIC*-rearranged sarcomas and HGNET fusion-positive is crucial, given that the former typically presents a worse prognosis [19]. This has been highlighted in the study by Philipp Sievers, which suggested the latter as intermediated malignancy [5]. Consequently, the accurate classification of sarcoma or neuroepithelial types of tumors harboring *CIC* fusions should be encouraged in routine diagnostics. Noteworthy, in our cohort, Case 2 and 3 demonstrated methylation profiles that were inconsistent with either *CIC*-rearranged sarcoma or HGNET *CIC* fusion-positive, but were closer to the MC of lower-grade entities. The outcome data appear to support their methylation signature: Case 2, despite displaying high-grade neuroepithelial tumor appearance, achieved a PFS of 56 months; Case 3 showed a lower-grade neuroepithelial tumor morphology with low Ki67 index. Without chemotherapy or radiotherapy, the patient has reached a PFS of 34 months post-GTR. The overall survival rate (Sup Fig. 1a) and PFS (Sup Fig. 1b) of CNS tumors with *CIC::LEUTX* fusions show better clinical outcomes compared to those with non-*LEUTX* fusions. However, given the limited cases numbers, more outcome data is needed to draw concrete conclusions.

These cases further indicate that the confirmation of *CIC*-rearranged sarcoma requires differential diagnosis of neuroepithelial tumors or rare lower-grade entities. Such differentiation should ideally integrate both morphological evidence and DNA methylation profiling rather than relying solely on the presence of *CIC* fusion alone, especially for those tumors harboring *CIC::LEUTX* fusion.

## Conclusion

In summary, our study expands the knowledge of *CIC*-rearranged pediatric CNS tumors, specifically those tumors harboring *CIC::LEUTX* fusions, which may be a heterogeneous group of tumors consisting of *CIC*-rearranged sarcomas, HGNET *CIC* fusion-positive, and rare lower-grade neuroepithelial tumors with undefined methylation signatures. The combination of GFAP, Olig-2, synaptophysin, WT-1 and reticulin staining can help differentiate sarcoma and neuroepithelial tumors. For unresolved cases, DNA methylation profiling serves as an ideal approach for precise and efficient classification. Studies on larger cohorts are still required for a better understanding these tumors.

### Electronic supplementary material

Below is the link to the electronic supplementary material.

Supplementary Material 1. **Supplementary Fig. 1**. Kaplan-Meier survival curve of overall survival rate**(a)**and progression-free survival **(b)** for CNS tumors with *CIC::LEUTX* and non-*LEUTX* fusions

## Data Availability

The datasets during and/or analysed during the current study available from the corresponding author on reasonable request.

## Abbreviations

*BRAF*: B-Raf Proto-Oncogene, Serine/Threonine Kinase
*CIC*: capicua transcriptional repressor
*DUX4*: double homeobox 4
ETMR: Embryonal tumor with multilayered rosettes
FFPE: Formalin-fixed paraffin-embedded
*FOXR2*: Forkhead box R2
GFAP: Glial fibrillary acidic protein
HGNET: high-grade neuroepithelial tumor
HPF: high power field
IHC: Immunohistochemistry
IDAT: Intensity data
*LEUTX*: leucine twenty homeobox
*MYB*: MYB Proto-Oncogene, Transcription Factor
NGS: Next-generation sequencing
NOS: not otherwise specified
*NUTM1*: NUT Midline Carcinoma Family Member 1
Olig-2: Oligodendrocyte transcription factor 2
WT-1: WT1 Transcription Factor
