# Supplementary figures and images for: Pediatric central nervous system tumor with CIC::LEUTX fusion: a diagnostic challenge
Source: Acta Neuropathol Commun. 2024 Jun 27;12:106. doi: 10.1186/s40478-024-01824-w (PMC11210039; doi:10.1186/s40478-024-01824-w)

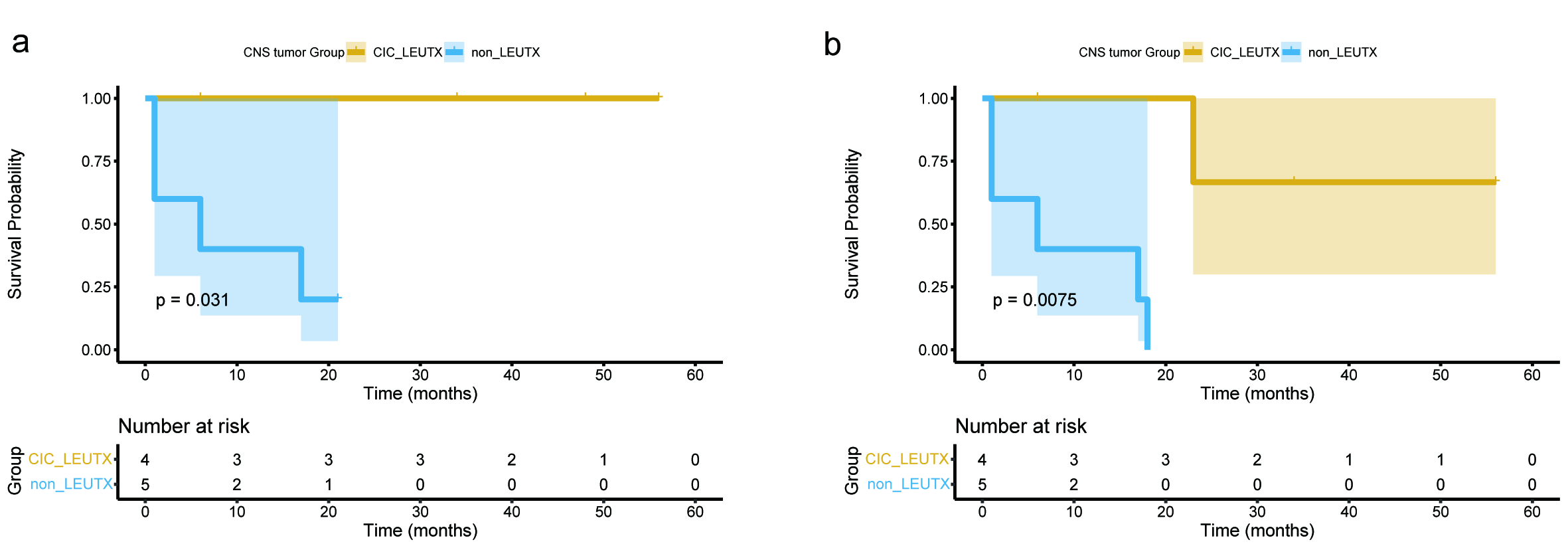

Supplement: Supplementary file 1 — Supplementary Material 1. Supplementary Fig. 1. Kaplan-Meier survival curve of overall survival rate(a)and progression-free survival (b) for CNS tumors with CIC::LEUTX and non-LEUTX fusions [file 40478_2024_1824_MOESM1_ESM.tif]
